# Iranians’ Perspective to Cosmetic Surgery: A Thematic Content Analysis for the Reasons

**DOI:** 10.29252/wjps.9.1.103

**Published:** 2020-01

**Authors:** Mitchell Brady

**Affiliations:** Idaho College of Osteopathic Medicine, Meridian, ID, USA

**Keywords:** Microsurgery training, Simulation model, Anastomosis

**DEAR EDITOR**

I have read the Original Article, “Iranians’ Perspective to Cosmetic Surgery: A Thematic Content Analysis for the Reasons” by Nahid et al. published in *The World Journal of Plastic Surgery *(Jan. 2019;8(1):69-77.). I would like to congratulate the Authors on these interesting findings. This study provided an insight into the internal motivations of facial surgery in Iranians, and I agree that this is a valuable insight to healthcare providers. It would be interesting to follow up with these individuals and see if the surgeries in fact impacted their lives as they believed they would. 

Did the men and women find more suitable husbands or wives? Did they gain more success? One women in the article prior to surgery failed to attract the opposite sex based on the shape of her nose.^1^ It would be interesting to see if her procedure has increased her success with attracting the opposite sex. It has been demonstrated that married women that underwent cosmetic plastic surgery had higher marital satisfaction than women that did not have cosmetic surgery.^2^ It would be interesting to see if there was any correlation.

## CONFLICT OF INTEREST

The authors declare no conflict of interest.
